# Glycoalkaloids Affect the Animal Nervous System: A Review

**DOI:** 10.1155/jt/1380303

**Published:** 2026-10-04

**Authors:** Gracjan Dudek, Aleksandra Jaworska, Paweł Marciniak

**Affiliations:** ^1^ Department of Animal Physiology and Developmental Biology Faculty of Biology Institute of Experimental Biology Adam Mickiewicz University Uniwersytetu Poznańskiego 6 Poznań 61-614 Poland amu.edu.pl

**Keywords:** anticancer activity, glycoalkaloids, neuroprotection, *Solanaceae*, toxicity

## Abstract

Glycoalkaloids (GAs) are plant secondary metabolites produced mainly by species of the *Solanaceae* family, such as the tomato *Solanum lycopersicum* L. Although traditionally associated with toxicity, recent research highlights their diverse biological activities, including anti‐inflammatory, anticancer, antimicrobial, and neuroprotective effects. These properties arise from their ability to interact with cell membranes, modulate key signaling pathways, and influence immune responses. Selected GAs, such as solasonine, solamargine, and α‐tomatine, have demonstrated the potential to regulate inflammation, induce apoptosis in cancer cells, affect the tumor microenvironment, and modulate microglial activity in the nervous system. Moreover, their toxicity remains an important limitation, emphasizing the need for further studies on structure–activity relationships and strategies to reduce adverse effects while preserving therapeutic potential. Overall, GAs represent promising candidates for future research aimed at developing novel therapeutic approaches, provided that their safety profiles are thoroughly characterized.

## 1. Introduction

Glycoalkaloids (GAs) are toxic steroidal alkaloids. They are widely distributed as natural secondary metabolites in plants of the *Solanaceae* family, including tomato (*Solanum lycopersicum*), pepper (*Capsicum annuum*), potato (*Solanum tuberosum*), and eggplant (*Solanum melongena*) [1]. Their content varies between individual plant organs and changes during ontogeny, with synthesis increasing under biotic and abiotic stress factors such as pathogenic bacteria and fungi, herbivorous insects, mechanical damage, and even natural aging [2].

The biosynthesis of GAs occurs in three stages. In the first two stages, the primary metabolites cycloartenol and cholesterol are formed, whereas in the third stage, the actual GAs are synthesized from cholesterol, their common precursor [3].

GAs are characterized by a chemical structure typical of heterocyclic bases containing a nitrogen atom. Their molecule consists of two parts: a nonsugar core, referred to as the aglycone, and a sugar moiety attached to it, known as the glycone. The aglycone forms the hydrophobic and nonpolar portion of the molecule and has a steroidal structure on the basis of a 27‐carbon cholestane skeleton with a nitrogen‐containing ring (Figure 1; colored). A sugar chain is attached to the hydroxyl group at the 3‐OH position, forming the hydrophilic part of the compound. The glycone may consist of three or four monosaccharides, such as D‐glucose, D‐galactose, L‐rhamnose, D‐xylose, or L‐arabinose, which can combine in various configurations [4]. For example, α‐solanine and α‐chaconine contain the same aglycone, solanidine, but differ in the structure of their sugar moieties. In α‐chaconine, a branched α‐chacotriose chain (bis‐α‐L‐rhamnopyranosyl‐β‐D‐glucopyranose) is attached to the 3‐OH group of the aglycone, whereas α‐solanine contains an α‐solatriose chain (α‐L‐rhamnopyranosyl‐β‐D‐glucopyranosyl‐β‐D‐galactopyranose), also linked to the same 3‐OH position of the steroidal core [2]. The letters α, β, and γ in the names of GAs indicate the presence of tri‐, di‐, or monosaccharide residues, respectively, which arise from the hydrolytic cleavage of glycosidic bonds within their structures [5].

**FIGURE 1:**
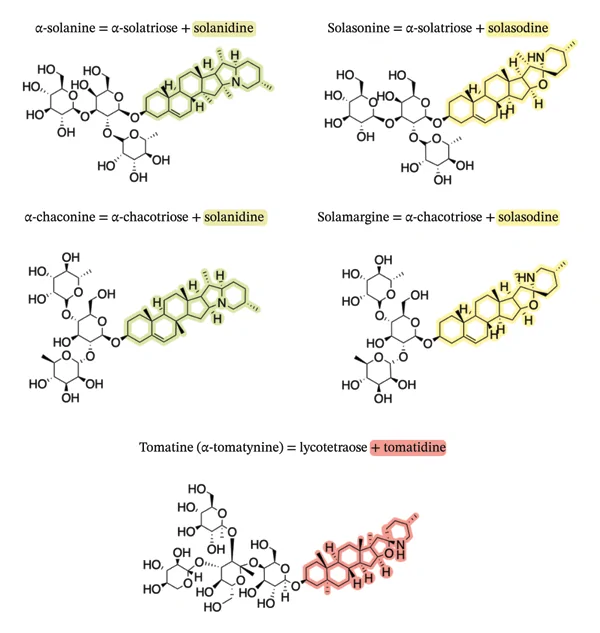
Glycoalkaloid structures.

This literature review was conducted as a narrative review using the Google Scholar, PubMed, Scopus and ScienceDirect databases. The literature search was performed mainly in English using combinations of keywords related to GA toxicity, its effects on the nervous system, mechanisms of action, and its potential anticancer and beneficial biological activities. The analysis focused on GAs occurring in plants of the *Solanaceae* family, with particular emphasis on α‐solanine, α‐chaconine, and α‐tomatine, as well as selected derivatives such as tomatidine, chaconine, and solamargine. Recent scientific publications were prioritized; however, older studies were also included when the reported findings remained relevant and scientifically valid.

## 2. Molecular Mechanisms of GA Action

It is suggested that the biological activity of GAs arises primarily from two properties [6].

a. Their ability to inhibit acetylcholinesterase (AChE) and butyrylcholinesterase (BuChE)
 Vertebrates possess two cholinesterases, AChE and BuChE, which differ in their kinetic properties, substrate and inhibitor specificity. AChE is found in neurons and erythrocytes, whereas BuChE is present in neurons, glial cells, and blood serum [7]. The inhibition of AChE was first reported in 1953 in early studies on *Solanaceae* GAs [8]. Once released into the synaptic cleft, acetylcholine binds to both nicotinic and muscarinic receptors on the postsynaptic membrane. This interaction opens ion channels and depolarizes the neuronal membrane while also altering the profile of secondary neurotransmitters [9–12]. AChE is responsible for degrading acetylcholine into acetate and choline. The released choline is then taken up and transported back to the axon, where it serves as a substrate for the resynthesis of acetylcholine [11]. Studies on the inhibition of AChE by GAs have been conducted in numerous animal species (Table 1) (Figure 2) [8].
 Three‐dimensional molecular docking analyses revealed that α‐tomatine forms a hydrogen bond of 2.6 Å with Trp86 in the active site of AChE, thereby inhibiting its activity and preventing the breakdown of acetylcholine [18]. In a recent in silico study by Idowu et al., the binding affinities of α‐chaconine and α‐solanine for AChE were also examined, and the authors reported that both compounds exhibit comparable binding energies [19]. Interestingly, not all GAs are capable of inhibiting AChE. Solamargine and solasonine—GAs found in eggplant—are examples of such compounds. Their aglycone, solasodine, determines whether binding to AChE is possible [20].
 In addition to AChE, BuChE also participates in the regulation of the cholinergic system [21]. BuChE shares 65% structural homology with AChE. The main difference between the two enzymes is the replacement of aromatic residues in the active site of AChE with aliphatic residues in BuChE, which reduces the efficiency of acetylcholine hydrolysis [22, 23]. However, studies have shown that mice in which the AChE gene is silenced are still able to move, breathe, and eat. These findings indicate that BuChE can compensate for the absence of AChE [24]. By binding to both BuChE and AChE, GAs impair their function, leading to the accumulation of acetylcholine in the synaptic cleft (Figure 2). This may manifest as impaired balance and motor coordination, shortness of breath, and tachycardia [4].
b. Their ability to form complexes with membrane 3β‐hydroxysterols, thereby disrupting the integrity and function of the cell membrane
 Because of their amphiphilic properties, GAs disrupt the structure of the cell membrane (Figure 2). The steroidal portion of the GA interacts with 3β‐hydroxysterols, such as cholesterol, causing deformation of the lipid bilayer and impairing its biological function. As a result, GAs increase membrane permeability, leading to leakage of cellular contents [2, 25–27]. Early observations of these effects were based on studies using Brewster angle microscopy [28]. Recent studies by Keuter and colleagues using the Caco‐2 cell line, derived from human colon adenocarcinoma, demonstrated that α‐chaconine, α‐tomatine, and solamargine act in a dose‐dependent manner. These compounds exhibit minimal membrane‐disrupting potential at a concentration of 1 μM but a strong effect at 10 μM. α‐Solanine had the weakest effect on the membranes at both concentrations, and the damaged cells retained the ability to regenerate their plasma membrane [29].
 In addition to the mechanisms described above, many studies focus on disruptions of intracellular signaling pathways induced by GAs. α‐Chaconine suppresses the phosphorylation of protein kinase B (Akt) and estrogen receptor α (ERα) in a dose‐dependent manner. In contrast, α‐solanine inhibited the phosphorylation of ERα only, and only at concentrations of 20 μM and 50 μM. In the same study, which was conducted using the RL95‐2 cell line (adenosquamous endometrial carcinoma), α‐chaconine and α‐solanine were shown to reduce the expression of Akt mRNA and ERα mRNA at concentrations of 5 μM and 30 μM, respectively [30]. This is particularly important because estrogen receptors are distributed randomly within the cell, and ligand binding leads to the dissociation of a corepressor protein and activation of the receptor. The phosphorylated dimers are then transported to the nucleus, where they regulate the transcription of specific target genes. A small fraction of receptors (approximately 10%) participate in nongenomic signaling by associating with the plasma membrane and activating various protein kinases, including protein kinase B, within the PI3K‐Akt‐mTOR pathway [31]. Protein kinase B can also directly phosphorylate ERα, inducing receptor activity independent of ligand binding. Deregulation of these pathways in many cancers results in increased cell proliferation and tumor growth, as well as enhanced migration and metastasis [32]. α‐Chaconine also suppressed the PI3K‐Akt and JNK pathways in studies using the BAEC cell line (bovine aortic endothelial cells). The observed effects were dose‐dependent (1–3 μg/mL) and time‐dependent (3–24 h) and correlated with reduced expression of matrix metalloproteinase (MMP‐2) and nuclear factor kappa‐light‐chain‐enhancer of activated B cells (NF‐κB) [33]. Both pathways disrupted in this study are involved in angiogenesis under physiological and pathological conditions, including tumor development [34, 35].
 In silico studies have shown that α‐solanine and α‐chaconine can bind to the adenosine A2A receptor (A2AR), with α‐chaconine binding more efficiently (Figure 2). Although the precise mechanism has not been experimentally confirmed, the compounds are suggested to act as agonists, leading to activation of the A2AR signaling pathway [36]. Activation of this pathway suppresses effector immune responses and stimulates immunosuppressive cell populations, thereby preventing excessive inflammatory reactions and protecting tissues from damage [37].

**TABLE 1:** Effects of glycoalkaloids on acetylcholinesterase and butyrylcholinesterase in various animal models.

Glycoalkaloid	Animal/tissue	Study type	Exposure route	Concentration or dose	Observed effect	Ref.
α‐Solanine	Sprague–Dawley rat (liver, brain, blood)	In vivo	Intraperitoneal	10 mg/kg b.w.	Inhibition of cholinesterase activity in the liver after 12 h; no significant effect on other tissues (brain, plasma, erythrocytes)	[13]

α‐Solanine	New Zealand rabbit (plasma, erythrocytes)	In vivo	Intraperitoneal	20–30 mg/kg b.w.	Effect more pronounced in plasma than erythrocytes; 30 mg/kg dose was lethal	[14]

α‐Solanine	Dog	In vivo	Intravenous	30 mg/kg b.w.	Initial serum AChE inhibition followed by rapid recovery; no impact on erythrocytes	

α‐Solanine+α‐chaconine (ratio 1:70)	Golden Syrian hamster	In vivo	Oral	33.3 mg/kg b.w.	Significant increase in brain AChE activity (∼10%) and modest increase in plasma BuChE	[15]

α‐Solanine	Human plasma and serum	In vitro	Direct incubation	0.083–1000 μM	Reversible inhibition of BuChE; chaconine is a more potent inhibitor; no inhibition at expected physiological blood levels	[16]
α‐Chaconine						

α‐Chaconine	Colorado potato beetle	In vitro	Homogenate incubation	1–40 μM	AChE inhibition; beetle enzyme showed significantly lower sensitivity to the glycoalkaloid than other insect species	[17]

α‐chaconine	Horse	In vitro	Direct incubation	4–30 μM	Inhibition of BuChE activity	

**FIGURE 2:**
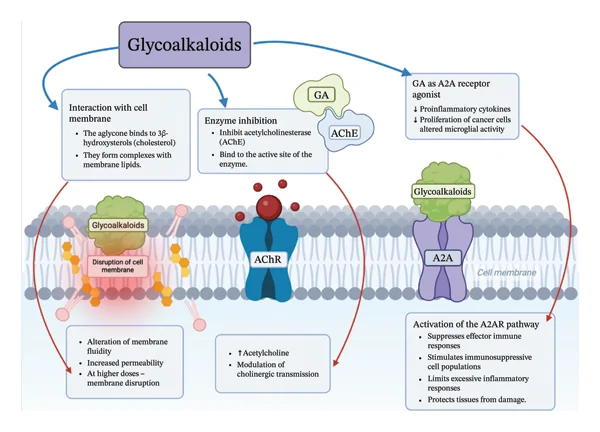
Molecular mechanisms of action of glycoalkaloids.

## 3. Adverse Effects of GAs

### 3.1. GA Poisoning

The properties of GAs have been intensively studied for several decades [38]. A wide range of animal models continue to be used to determine not only their toxicity but also the mechanisms underlying their biological effects [8]. The toxicity of a given GA depends on the route of administration. Compared with oral administration, significantly lower doses produce symptoms—or can be lethal—when administered intravenously or intraperitoneally [39]. At lower doses, GAs primarily cause gastrointestinal disturbances such as vomiting, diarrhea, and abdominal pain. At higher doses, more severe symptoms, including fever, tachycardia, hypotension, and neurological disturbances, may occur [40, 41]. Severe GA poisoning may lead to paralysis, respiratory failure, cardiac failure, coma, or death [42]. Symptoms typically appear 2–4 h after a single exposure and may persist for 8 to 24 h [43].

The toxicity of a compound also depends on the animal species used in the study. For example, the LD_50_ values determined for α‐solanine (a GA derived from the potato *Solanum tuberosum*) administered orally ranged from 32.3 to 42 mg/kg body weight in mice, whereas in rats, the value was 67 mg/kg. Another potato GA, α‐chaconine, had LD_50_ values of 19.2–32.3 mg/kg in mice and 84 mg/kg in rats [39]. For most animals, the LD_50_ of other GAs falls within the range of 30–60 mg/kg body weight [44]. To protect consumers, the GA content in potato tubers should be < 20 mg per 100 g of fresh weight [40]. In 2020, the European Food Safety Authority (EFSA), through its CONTAM panel on food chain contaminants, established a new lowest‐observed‐adverse‐effect level (LOAEL) for humans of 1 mg/kg body weight. Daily exposure to 3–6 mg/kg body weight of total potato GAs is considered potentially lethal [45].

GAs, whether administered orally, intraperitoneally, or intravenously at sufficient concentrations, can cause death in many animal species, including sheep, rats, mice, rabbits, and rhesus macaques [41, 46]. Pregnant animals are more sensitive to α‐chaconine than nonpregnant animals, even when the administered dose is reduced by half. Rat fetuses are up to twice as sensitive as their mothers are as follows: doses of α‐solanine that cause 18%–100% fetal mortality (80 and 160 mg/kg body weight) have no effect on mothers [47].

### 3.2. Effects of GAs on the Animal Nervous System

GAs—particularly α‐solanine—were studied even more intensively after Renwick proposed in 1972 a hypothesis linking potato tubers to defects in the developing neural tube [48]. Developmental studies conducted in pregnant Syrian hamsters (*Mesocricetus auratus*) exposed to GAs have also described their effects on the central nervous system, including defects such as anophthalmia, exencephaly, and encephalocele [39, 49]. In vitro studies on bovine embryos revealed that GAs reduce embryonic developmental rates, with α‐solanine and α‐chaconine exhibiting a strong synergistic effect that results in developmental abnormalities and increased mortality [50]. Similar conclusions were drawn by Rayburn and colleagues in studies on the toxicity of *Solanaceae* GAs in *Xenopus laevis* embryos [51].

Neural tube defects (NTDs), including exencephaly as a form of anencephaly—the most severe type of NTD—or encephalocele, arise from improper closure of the neural tube [52, 53]. They occur primarily sporadically, with environmental and genetic factors contributing to their etiology. In the presence of NTDs, the cranial bones are malformed, and the brain and cerebellar structures are reduced or severely distorted [54]. In an extensive study, Ni and colleagues analyzed the relationship between the consumption of sprouting potatoes and the risk of NTDs (anencephaly, encephalocele, and spina bifida) and orofacial clefts (OFCs). They confirmed the teratogenic properties of α‐solanine and reported that vitamin B intake is a beneficial factor that counteracts the occurrence of both NTDs and OFCs [55]. In contrast to these findings, Ekmekci and colleagues, in a recent study, did not demonstrate an association between solanine levels in serum or amniotic fluid and the occurrence of anencephaly in human fetuses [52].

The effects of GAs on insects have also attracted scientific interest, particularly among researchers seeking alternatives to conventional insecticides with reduced environmental and ecological impact [56]. Plant‐derived compounds can influence the neuroendocrine system [57]. The retrocerebral complex—a key structure of the insect neuroendocrine system that is functionally comparable to the hypothalamic–pituitary axis in vertebrates—undergoes deformation after administration of α‐solanine and α‐tomatine to the beetles *Tenebrio molitor* and *Zophobas atratus* [58, 59]. These structural alterations may underlie developmental and reproductive disturbances in insects, for example, through dysfunction of the *corpora allata*, a component of the retrocerebral complex responsible for the synthesis and secretion of juvenile hormone [60].

## 4. Beneficial Effects of GAs

### 4.1. Anti‐Inflammatory Activity

Research on anti‐inflammatory activity is of key importance, as chronic inflammation is a major factor underlying the development and progression of many diseases, including cancer, neurodegenerative disorders, metabolic diseases, and autoimmune conditions. Prolonged activation of inflammatory pathways leads to cellular and tissue damage, disruption of systemic homeostasis, and an increase in oxidative stress, which further amplifies pathological processes (Figure 3) [25, 61]. For this reason, identifying compounds capable of modulating inflammatory responses is crucial both for prevention and for the development of new therapeutic strategies. In this context, GAs are also being investigated. Experimental studies have shown that solasonine reduces the expression of pro‐inflammatory mediators in U87 MG glioma cells in a dose‐dependent manner. This effect is reflected by decreased mRNA levels of interleukin‐6 (IL‐6) and tumor necrosis factor‐α (TNF‐α) and reduced protein levels of inducible nitric oxide synthase (iNOS) and cyclooxygenase‐2 (COX‐2) [62]. Moreover, solasonine inhibited the activation of the transcription factor NF‐κB by limiting the translocation of its p50/p65 subunits from the cytoplasm to the nucleus, leading to reduced expression of genes associated with inflammatory responses and glioma cell survival [62]. In the same model, a decrease in the phosphorylation of p38 and JNK kinases was also observed, indicating the attenuation of MAPK‐dependent inflammatory signaling [62]. An opposite effect was observed for α‐chaconine, which exacerbated pathological processes in an osteoarthritis model. This compound activated the NF‐κB pathway, as evidenced by increased phosphorylation of the IκBα inhibitor and nuclear translocation of the p65 subunit. This effect was associated with the induction of chondrocyte pyroptosis and the worsening of degenerative changes [63].

**FIGURE 3:**
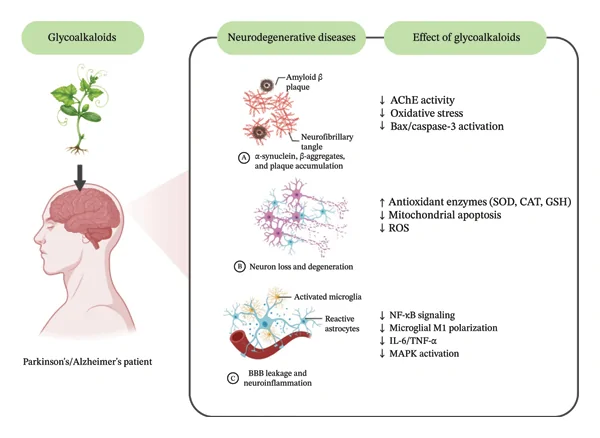
Effects of glycoalkaloids on the nervous system in neurodegenerative diseases.

### 4.2. Antibacterial and Antiviral Activity

GAs exhibit antifungal, antiviral, and antibacterial activity [58]. These microbial populations form the microbiome not only of humans but also of other animals [64]. From an immunological perspective, microorganisms are recognized as pathogens by the host immune system, which identifies and eliminates them. However, most intestinal bacteria are nonpathogenic and coexist with the host, assisting in nutrient metabolism, drug metabolism, and protection against colonization by pathogenic microbes [65]. It is estimated that the gut microbiota comprises at least 1800 genera and 15,000–35,000 bacterial species [66]. A healthy intestinal microbiota is dominated by two major phyla, *Firmicutes* and *Bacteroidetes*, with *Proteobacteria*, *Actinobacteria*, and *Fusobacteria* represented to a lesser extent [67]. The gut microbiome can significantly influence many aspects of physiology, including brain function and behavior [68]. The gut–brain axis is a bidirectional communication pathway that operates through neuronal, hormonal, and immunological mechanisms [69]. Potato extracts containing GAs exhibit antibacterial activity against Gram‐positive bacteria, including *Firmicutes*, *Actinobacteria*, and *Lactobacillus* [70]. One might expect such activity to weaken the gut microbiota, yet studies using a porcine cecum model indicate that GAs—α‐solanine, α‐chaconine, and α‐tomatine—can modulate the composition of the intestinal microbiome, for example, by improving the Bacteroidetes to Firmicutes ratio, which is considered beneficial for health [71]. Moreover, studies have shown that α‐tomatine does not inhibit the growth of nonpathogenic Lactobacillus or *Escherichia coli* strains present in the gut, suggesting the potential to combine antibacterial properties with preservation of the intestinal microbiota [72].

Tomatidine, the aglycone of α‐tomatine, also has notable antiviral properties [73]. Studies suggest that tomatidine acts against all four serotypes of dengue virus (DENV). It suppresses the expression of the cellular factor activating transcription 4 (ATF4), resulting in a greater than 90% reduction in viral replication, although the precise mechanism remains unknown [74]. A global increase in dengue incidence has been observed, along with a rise in neurological complications. These complications span a wide spectrum, including headache, seizures, hemiparesis, and even coma [75].

### 4.3. Anticancer Activity

Cancers are characterized by dysregulated processes such as proliferation, apoptosis, cell migration, and immune responses, which is why compounds capable of simultaneously modulating multiple signaling pathways are of particular interest. Owing to their specific chemical structure and ability to interact with cell membranes and key molecular regulators, steroidal GAs exhibit a broad spectrum of biological activities in cancer models [62, 76]. Tomatine reduced cell viability and increased the proportion of apoptotic cells in metastatic melanoma cell lines at concentrations corresponding to 0.5–1 µM α‐tomatine [77]. Tomatine strongly inhibited melanoma cell invasiveness in the Boyden chamber assay, reducing invasion by approximately 30%–35% at 0.5 µM and by approximately 65%–80% at 1 µM α‐tomatine [77]. Tomatine also reduced melanoma‐dependent angiogenesis, which the authors attributed to decreased vascular endothelial growth factor (VEGF) release and diminished stimulation of vascular‐like structure formation in the Matrigel assay [77]. Solasonine inhibited the proliferation, migration, and colony formation of glioma cells and induced apoptosis through a mitochondrial mechanism, involving cytochrome c release, an increased Bax/Bcl‐2 ratio, and the activation of caspases‐3/9 and PARP (poly (ADP‐ribose) polymerase) [78]. In hepatocellular carcinoma (HCC) cells, solamargine inhibited proliferation and induced apoptosis and autophagy both in vitro and in vivo, with the LIF/miR‐192‐5p/CYR61/Akt axis identified as a key mechanism [79]. Solamargine also modulated the tumor microenvironment in HCC by repolarizing M2 macrophages toward an M1‐like phenotype and influencing other immune cell populations within the tumor, which the authors associated with its anticancer activity (Figure 3) [79]. In studies using the SH‐SY5Y neuroblastoma cell line, solamargine induced apoptosis, with an IC_50_ of 15.62 µM. The authors suggested that solamargine may trigger chromatin condensation and fragmentation, similar to the effects observed in Hep3B liver cancer cells [80, 81]. A study by Santonocito and colleagues also confirmed the ability of α‐tomatine and tomatidine to reduce neuroblastoma cell populations, establishing optimal effective concentrations of 0.25 µg/mL for α‐tomatine and 0.50 µg/mL for tomatidine [82].

### 4.4. Neuroprotective Activity

In the SH‐SY5Y neuroblastoma cell model, α‐tomatine has neuroprotective effects, shielding cells from hydrogen peroxide‐induced neurotoxicity. This effect is reflected by increased cell viability and reduced membrane damage, as assessed by decreased lactate dehydrogenase (LDH) release following preincubation with tomatine [83]. Additionally, tomatine restored the activity of antioxidant enzymes suppressed by H_2_O_2_, including superoxide dismutase, catalase, and glutathione peroxidase, as well as the level of reduced glutathione. This was identified as a key mechanism underlying its protective action. In the same model, tomatine reversed the decrease in brain‐derived neurotrophic factor (BDNF) expression, indicating the involvement of this neurotrophic molecule in the protective effects of tomatine [83]. Furthermore, this GA reduced the expression of Bax (Bcl‐2‐associated X protein) (Figure 3) and inhibited the activation of caspase‐9 and caspase‐3 under oxidative stress conditions, which is consistent with the suppression of the mitochondrial apoptotic pathway in neuronal cells [83].

In silico studies on Parkinson’s disease have indicated that tomatidine has the potential to inhibit the enzyme LpxC (which is involved in LPS synthesis) and to block the TLR4 receptor by binding to the MD‐2 protein [84]. In a rat model of Parkinson’s disease, treatment with α‐solanine and α‐chaconine at a dose of 0.06 mg/kg body weight increased acetylcholine and GABA levels and reduced S100β protein levels, thereby decreasing inflammation and the compensatory response in the brain [85]. Comparable levels of neurotrophic factors in samples from rats treated with pramipexole (a drug used in advanced Parkinson’s disease) and those treated with GAs further support their neuroprotective effect [85]. In the same study, the authors reported that GA treatment did not affect dopamine or serotonin levels [85].

In individuals with Alzheimer’s disease, AChE activity increases, leading to increased breakdown of acetylcholine and a consequent reduction in its levels in the brain. AChE is also involved in the formation of β‐amyloid plaques, which significantly contribute to disease progression [86]. AChE inhibitors are standard agents used in the pharmacotherapy of Alzheimer’s disease [87]. GAs such as α‐solanine and α‐chaconine act as AChE inhibitors in the nervous system (Figure 3) [4]. Compounds with such properties have potential therapeutic applications [88].

Recent studies have shown that tomatidine alleviates stroke‐induced cognitive impairment (PSCI) and neurodegeneration [89]. Up to 38% of stroke survivors experience cognitive impairment 1 year after the event; 10% develop dementia within the first year, and within 3 years, another quarter of patients are affected [90]. The development of PSCI, however, results from multiple factors, including distorted and dysfunctional blood vessels as well as neurodegenerative processes, including chronic inflammation [91, 92]. In mouse studies, tomatidine was shown to reduce the expression of M1 microglial markers associated with inflammation, likely through modulation of the NF‐κB pathway, making tomatidine a promising candidate for the development of new strategies to prevent PSCI (Figure 3) [89].

## 5. Perspectives

Recent studies on GAs have employed multiple experimental approaches, including in vivo studies in rodent models, in vitro analyses performed using cellular models, and in silico approaches aimed at predicting molecular interactions and biological effects [2]. The heterogeneity of reported findings, and in some cases apparent inconsistencies between studies, reflects the multifaceted nature of GA activity and highlights the complexity of the underlying molecular mechanisms. These observations further emphasize the need for continued research to elucidate the biological roles, toxicological properties, and therapeutic potential of these compounds [8]. To facilitate the interpretation of the current evidence, Table 2 summarizes the principal biological activities of GAs, the experimental models in which they have been investigated, and their reported biological effects, highlighting both their toxicological properties and their therapeutic potential.

**TABLE 2:** General biological activity of glycoalkaloids (in vivo and in vitro).

Glycoalkaloid	Model/animal	Dose/concentration	Test/method	Biological effect	Ref.
α‐Solanine	Mouse (in vivo)	LD_50_: 32.3–42 mg/kg b.w.	Acute oral toxicity assay	Mortality/determination of lethal dose	[39]

α‐Solanine	Rat (in vivo)	LD_50_: 67 mg/kg b.w.	Acute oral toxicity assay	Mortality/determination of lethal dose	[39]

α‐Solanine	Pregnant rats/fetuses (in vivo)	80 and 160 mg/kg b.w.	Fetal mortality and teratogenicity evaluation	18−100% fetal mortality with no observed effect on mothers	[47]

α‐Solanine	Syrian hamster *(Mesocricetus auratus)* (in vivo)	70–150 and 200 mg/kg b.w.	Developmental neural tube defect assessment	Neural tube defects (anophthalmia, exencephaly, encephalocele)	[49]

α‐Solanine, α‐tomatine	Beetles (*T. molitor*, *Z. atratus*) (in vivo)	0.12–0.14 ng/mg b.w.	Histological examination of the neuroendocrine system	Deformity and structural alteration of the retrocerebral complex	[58, 59]

α‐Chaconine	Mouse (in vivo)	LD_50_: 19.2−32.3 mg/kg b.w.	Acute oral toxicity assay	Mortality/determination of lethal dose	[39]

α‐Chaconine	Rat (in vivo)	LD_50_: 84 mg/kg b.w.	Acute oral toxicity assay	Mortality/determination of lethal dose	[39]

α‐Chaconine	Chondrocytes (osteoarthritis model) (in vitro)	10 and 20 mg/kg b.w. MTT assay	NF‐κB signaling and IκBα phosphorylation assay	Induction of chondrocyte pyroptosis and aggravation of degenerative changes	[63]

α‐Chaconine/α‐solanine	RL95‐2 cell line (endometrial carcinoma) (in vitro)	5 μM (α‐chac.), 20–50 μM (α‐sol.)	mRNA expression analysis (RT‐qPCR) and protein phosphorylation (Western blot)	Inhibition of Akt and Erα phosphorylation; downregulation of Akt and Erα mRNA	[30]

α‐Chaconine	BAEC cell line (bovine aortic endothelial cells) (in vitro)	1–3 μg/mL	Gelatin zymography (MMP‐2) and PI3K/Akt/JNK pathway expression analysis	Suppression of angiogenesis; reduction of MMP‐2 and NF‐κB expression	[33]

α‐Solanine + α‐chaconine	Rats (Parkinson’s disease model) (in vivo)	0.06 mg/kg b.w.	Brain neurotransmitter quantification and neurodegeneration marker measurement	Increased acetylcholine and GABA; decreased S100β protein; neuroprotective effect	[85]

α‐Solanine + α‐chaconine	Bovine embryos (in vitro)	0.1–10.0 μM	in vitro embryonic development rate assay	Reduced developmental rate, developmental abnormalities, increased mortality (synergistic effect)	[50]

α‐Solanine + α‐chaconine	*Xenopus laevis* embryos (in vitro)	LC_50_ (96 h) EC_50_ (96 h) TI > 1.5	Frog Embryo Teratogenesis Assay—Xenopus (FETAX)	Synergistic developmental toxicity and teratogenicity	[51]

Solasonine	U87 MG cell line (glioma) (in vitro)	5–30 mg/kg b.w. IC_50_ 24–48 h CCK‐8/MTT	RT‐qPCR (IL‐6, TNF‐α) and Western blot (iNOS, COX‐2, NF‐κB p50/p65 translocation)	Anti‐inflammatory effect; suppression of pro‐inflammatory mediators and NF‐κB activation	[62]

Solasonine	Glioma cells (in vitro)	10 and 20 mg/kg b.w. IC_50_: 3.97 M (U87 MG), 6.68 M (U251), and 13.72 M (U118 MG)	Proliferation, migration, colony formation, and apoptosis assays (caspases‐3/9, PARP, Bax/Bcl‐2)	Induction of mitochondrial apoptosis; inhibition of proliferation and migration.	[78]

Solamargine	Caco‐2 cell line (colon adenocarcinoma) (in vitro)	1 and 10 μM	Plasma membrane integrity and permeability assay	Minimal effect at 1 μM; strong membrane‐disrupting effect at 10 μM	[29]

Solamargine	Hepatocellular carcinoma (HCC) cells (in vitro/in vivo)	5 and 10 mg/kg b.w. CCK‐8	Apoptosis, autophagy, macrophage repolarization assays (M2 to M1), and xenograft model	Inhibition of proliferation, induction of apoptosis/autophagy, tumor microenvironment modulation.	[79]

Solamargine	SH‐SY5Y cell line (neuroblastoma) (in vitro)	IC_50_: 15.62 μM	Cell cytotoxicity assay and nuclear morphology assessment	Apoptosis induction; chromatin condensation and fragmentation.	[80, 81]

α‐Tomatine	Metastatic melanoma cell lines (in vitro)	0.5−1 μM	Cell viability, Boyden chamber invasion, and Matrigel angiogenesis assays	Reduced viability; apoptosis induction; reduced invasion (30−80%) and VEGF release.	[77]

α‐Tomatine/tomatidine	Neuroblastoma cell lines (in vitro)	0.25 μg/mL (α‐tomatine), 0.50 μg/mL (tomatidine)	Cell viability/cytotoxicity assay	Reduction of neuroblastoma cell populations.	[82]

α‐Tomatine	SH‐SY5Y cell line (H_2_O_2_‐induced oxidative stress) (in vitro)	PC‐3 cells 0.5, 1.0 and 2.0 μM LNCaP cells 0.25–1.0 μM	LDH release, antioxidant enzymes (SOD, CAT, GSH), BDNF expression, and caspase‐3/9 assays	Neuroprotection: decreased LDH leakage; restored antioxidant enzymes and BDNF; inhibited apoptosis.	[83]

Tomatidine	Dengue virus (DENV) (in vitro)	0.1–10 μM EC_90_ = 1.61 μM SI = 97.7	Viral replication assay and ATF4 cellular factor expression measurement	Antiviral activity: suppression of ATF4; > 90% reduction in viral replication.	[74]

Tomatidine	Mice (poststroke cognitive impairment model‐PSCI) (in vivo)	10 and 20 mg/kg b.w. 1–10 μM (e.g., 2.5, 5, 10 μM) CCK‐8, MTT	Behavioral cognitive testing and M1 microglial inflammatory marker analysis	Alleviation of cognitive impairment; reduced M1 microglial markers via NF‐κB pathway modulation	[89]

Potato extracts	Gram‐positive bacteria (in vitro)	10–500 μM MIC = 25–100 μg/mL MTT, MTS	Antimicrobial susceptibility testing (Zone of inhibition/MIC)	Antibacterial activity against Gram‐positive bacteria.	[70]

α‐Solanine, α‐Chaconine, α‐Tomatine	Porcine cecum model (ex vivo)	10 μM	Gut microbiome composition analysis (16S rRNA gene sequencing)	Modulation of gut microbiota composition (improved *Bacteroidetes*‐to‐*Firmicutes* ratio).	[71]

On the basis of the current research, several realistic directions for further investigation into GAs and their potential therapeutic applications can be identified [8]. An important area involves deepening the understanding of structure–activity relationships (SAR). Modifying the sugar moiety of the molecule could reduce toxicity while preserving anticancer or anti‐inflammatory activity [4]. Moreover, the aglycone strongly influences the ability to interact with specific enzymes, such as AChE, which suggests the possibility of designing compounds with more targeted effects—for example, neuroprotective effects rather than neurotoxic effects [62]. In the context of the nervous system, findings on tomatine and tomatidine are particularly noteworthy, as they modulate microglial activity through effects on the NF‐κB pathway. These findings suggest their potential use in preventing cognitive impairment following stroke [89]. Additionally, their ability to inhibit AChE indicates the potential for further research into neurodegenerative diseases such as Alzheimer’s disease [88]. In oncology research, GAs simultaneously affect proliferation, apoptosis, angiogenesis, and components of the tumor microenvironment [76]. These findings suggest that these compounds could be considered adjuvant compounds in anticancer therapy, enhancing tumor cell sensitivity to other drugs or modulating immune responses, for example, by influencing macrophage polarization [79]. Ultimately, research on GAs aims not only to determine their toxicity but also to identify a safe range of biological activity and to assess whether plant‐derived compounds can serve as starting points for the development of new therapeutic strategies [4].

## Author Contributions

Gracjan Dudek: conceptualization and writing–original draft; Aleksandra Jaworska: writing–original draft and visualization; Paweł Marciniak: supervision and writing–review and editing.

## Funding

This work was supported by Grant No. 2023/51/B/NZ3/01550 from the National Science Centre (Poland) awarded to Paweł Marciniak.

## Conflicts of Interest

The authors declare no conflicts of interest.

## Data Availability

Data sharing is not applicable to this article as no datasets were generated or analyzed during the current study.
